# Folic Acid Alleviates High Glucose and Fat-Induced Pyroptosis via Inhibition of the Hippo Signal Pathway on H9C2 Cells

**DOI:** 10.3389/fmolb.2021.698698

**Published:** 2021-10-07

**Authors:** Lei Hong, Yingjie Zha, Chen Wang, Shigang Qiao, Jianzhong An

**Affiliations:** ^1^ Institute of Clinical Medicine Research, Affiliated Suzhou Science and Technology Town Hospital of Nanjing Medical University, Suzhou, China; ^2^ Department of Anesthesiology, Affiliated Suzhou Science and Technology Town Hospital of Nanjing Medical University, Suzhou, China

**Keywords:** type 2 diabetes, diabetic cardiomyopathy, folic acid, pyroptosis, hippo signal pathway

## Abstract

Diabetic cardiomyopathy (DCM) is the leading cause of death in diabetic patients. Folic acid has a protective effect on diabetes-induced cardiomyocyte damage. The aim of this study was to explore the effects of folic acid on cardiomyocytes cultured under high glucose and fat (HGF) conditions and type 2 diabetes mellitus (T2DM) mice, and elucidate the underlying mechanisms. Bioinformatics analysis was used to identify the potential drugs through the Drug-Gene Interaction database. H9C2 cardiomyocytes were cultured with 30 mM glucose and 500 nM palmitic acid in the presence or absence of folic acid or YAP1 inhibitor (verteporfin) or YAP1 siRNA. The cell viability and lactate dehydrogenase (LDH) release were measured using specific assay kits. Pyroptosis was detected by flow cytometry. The concentrations of IL-1β and IL-18 in the supernatants were measured by ELISA. The NLRP3, ASC and caspase-1 mRNA levels were detected by qRT-PCR and that the proteins expression of NLRP3, ASC, cleaved caspase-1 (p10), caspase-1, YAP1, p-YAP1, LATS1 and P-LATS1 were detected by Western blotting. C57BL/6 mice were fed with high fat diet (HFD) combined with streptozotocin (STZ) intraperitoneally to establish a T2DM model, folic acid or PBS treatment for 8 weeks by oral gavage, blood glucose and body weight were measured every 4 weeks, mouse heart tissue was used to detect pyroptosis and hippo signaling pathway related protein expression. We identified 427 differentially expressed genes in the cardiac tissues of high fat diet + streptozotocin mice, among the 30 most significantly DEGs, folic acid was predicted to be the most likely therapeutic drug. Folic acid alleviated HGF-induced cell damage *in vitro* and *in vivo* by decreasing activation of the Hippo pathway, as indicated by lower LDH release and increased cell viability, and decreased expression of NLRP3, ASC, cleaved caspase-1, IL-1β, IL-18, p-YAP and p-LATS. Verteporfin or YAP1 siRNA neutralized the protective effect of folic acid by reversing YAP1-induced pyroptosis. Folic acid reduced NLRP3 inflammasome-mediated pyroptosis by down-regulating the Hippo signaling pathway, thereby effectively reducing T2DM-induced damage in H9C2 cells and animals.

## Introduction

Diabetes mellitus is the most prevalent metabolic disorder globally and is associated with considerable socio-economic burden. Around 90–95% of the diabetics have type 2 diabetes mellitus (T2DM) that is characterized by insufficient insulin secretion by pancreatic *β*-cells, which leads to insulin resistance (Roden and Shulman, 2019). T2DM is often accompanied by severe chronic complications, including neuropathies, nephropathies, cardiovascular and cerebrovascular diseases (Artasensi et al., 2020; Peiris, 2020; Sachinidis et al., 2020). Diabetic cardiomyopathy (DCM) is characterized by aberrant changes in the cardiac structure and function, and is one of the main causes of death in diabetic patients (Ward and Crossman, 2014). Cardiomyocytes frequently show a significant increase in the surface area and hypertrophy, which lead to significant cellular damage and death in the late stage of T2DM (Adderley et al., 2020). In addition, a hyperglycemic environment also promotes cardiac fibroblasts (CFs) dysfunction, excessive fibrosis, and imbalance between various collagen types (Filardi et al., 2019). The Hippo signaling pathway is closely related to cell proliferation and death. High glucose levels increased phosphorylation of the Hippo pathway mediators including mammalian sterile 20-like kinase 1 (MST1) and large tumor suppressor 1 (LATS1), along with the nuclear translocation of Yes-associated protein (YAP) in the CFs, which translated to increased proliferation and invasion, collagen production and inflammation (Liu et al., 2020). Endothelial-specific MST1 transgenic mice exhibited worse cardiac function and aggravated insulin resistance compared to non-transgenic diabetic mice (J. Hu et al., 2018). One study showed that folic acid improved retinal vascular endothelial injury through the Hippo signaling pathway by downregulating p-YAP1 and p-LATS (Gorabi et al., 2020) (Z Wang et al., 2018).

Multiple mechanisms were involved in the development of DCM, including inflammation (Y Chen Y. et al., 2020) and the inflammatory form of programmed cell death known as pyroptosis (Chen, et al., 2020b). Hyperglycemic stimulation activated the nod-like receptor with pyrin domain containing 3 (NLRP3) inflammasome, which culminated in the activation of caspase-1, and maturation of the pro-inflammatory cytokines IL-1β and IL-18, resulting in pyroptosis and DCM in diabetic mice. Thus, inactivation of the NLRP3 inflammasome is a rational approach for improving heart function during diabetes (Chen, et al., 2020a).

Folic acid (vitamin B9) is essential for DNA/RNA synthesis, and its deficiency is associated with a higher risk of neural tube defects, cancer and coronary heart disease (Z. Wang et al., 2018; Lachenauer et al., 2020; Jelicic et al., 2020). Previous studies have shown that the severity of diabetic retinopathy is inversely correlated to circulating folic acid levels (Malaguarnera et al., 2015). Folic acid supplementation effectively reduced the fasting blood glucose levels, insulin resistance index and blood insulin levels in diabetic patients, indicating its potential therapeutic value in diabetes (Dierkes et al., 2018). In addition, folic acid supplements improved endothelial dysfunction in T2DM patients, regardless of the reduced homocysteine levels and inflammation markers (Malaguarnera, et al., 2015). There is also evidence indicating a protective effects of folic acid on cardiovascular diseases in T2DM (Mutavdzin et al., 2019), although its mechanistic role in cardiomyocytes is still unclear.

In this study, we identified five hub genes (Ceacam1, Art4, Vnn1, Ntm, and Folr2) associated with T2DM through bioinformatics analysis (data not shown). Ceacam1 is related with insulin resistance, and its liver-specific deletion can lead to chronic hyperinsulinemia, impaired insulin clearance, hepatic insulin resistance and steatosis (Ghadieh et al., 2019). Vanins (Vnn) are enzymes that convert pantetheine to vitamin B5, and while absence of Vnn1 activity improved insulin sensitivity in high fat diet-fed animals, its short-term inhibition may have limited value as an anti-diabetic strategy (van Diepen et al., 2016), neurotrimin (Ntm) is a novel biomarker of heart failure (Cao et al., 2015). In addition, folic acid was predicted as a potential drug for DCM through the Drug-Gene Interaction database. Our research aim is to explore whether folic acid may inhibit T2DM induced pyroptosis through the Hippo pathway.

## Methods and Materials

### Data Preprocessing and Identification of DEGs

The cardiac mRNA expression profiles of 3 ExVivoLV_non-T2DM and 3 ExVivoLV_T2DM rats calculated on the Agilent-028282 Whole Rat Genome Microarray 4 × 44K v3 platform were derived from the GEO dataset GSE99411. The differentially expressed genes (DEGs) were calculated by the Limma package using absolute log2 fold change (FC) > 1 and *p* value <0.05 as the cutoff criteria, and corrected by the Benjamini–Hochberg method.

### Drug-Gene Interaction

Potential new drugs or compounds for treating DCM were identified from the Drug-Gene Interaction database (DGIdb; http://www.dgidb.org/search_interactions) based on the essential genes.

### Cell Culture

The H9C2 cells were purchased from Shanghai GeneChem Co. Ltd. (Shanghai, China). The cell lines were maintained in Dulbecco’s modified Eagle’s medium (DMEM, HyClone, United States) supplemented with 10% fetal bovine serum (FBS, Gbico, United States), 100 U/ml penicillin and 100 µg/ml streptomycin (Beyotime Biotechnology, Shanghai, China) at 37°C in a 5% CO_2_ humidified atmosphere. As per the experiment, the cells were divided into five groups: cultured with DMEM medium as the control group (NG), 30 nM glucose and 500 nM palmitic acid as high fat and high glucose (HGF) group (Sohlang and Majaw, 2021), HGF and 5, 50 or 500 nM folic acid (Sigma, 59303, Germany).

### CCK-8 Assay

The CCK-8 assay was performed to assess the cytotoxicity of folic acid and verteporfin (a YAP inhibitor) according to the manufacturer’s instructions. Briefly, H9C2 cells were seeded into 96-well plates at the density of 5,000 cells per/well and cultured overnight. Following treatment with different concentrations of folic acid (0, 5, 50, 500 nM) or verteporfin (0, 1.25, 2.5, 5, 10, 20 μM) for 48 h, the medium was replaced with 100 µl fresh medium and 10 µl CCK-8 reagent (Dojindo Laboratories, Kumamoto, Japan) per well. The plates were incubated at 37°C for 2 h, and the OD at 450 nm was measured using a microplate reader (Tecan, Switzerland).

### LDH Measurement

Lactate dehydrogenase (LDH) levels in cellular supernatants were measured using an LDH assay kit (Jian-cheng, Nanjing, China) according to the manufacturer’s instructions.

### Flow Cytometry Assay

Pyroptosis was evaluated by flow cytometry (Agilent Technologies, United States) using the FAM-FLICA Caspase-1 Kit (ImmunoChemistry Technologies, LLC, 9,161, United States) according to the manufacturer’s instructions, PI staining was used to detected the dead cells. Single-stained tubes were used for calibration, while double-stained tubes were used to analyze the proportion of pyroptosis cells in the sample to be tested, the caspase-1 (FITC) and PI (PE) positive cells were regard as pyroptosis cells. In addition, Annexin V-PE/7-ADD (eBioscience, Cat#559763, United States) was detected by flow cytometry to measure death ratio of H9C2. In brief, cells were collected, washed twice with cold PBS, and then incubated with Annexin V and 7-AAD for 15 min. Finally, Annexin V and 7-AAD both positive cells was regarded as dead cells.

### RNA Interference

H9C2 cells were infected with a YAP1 siRNA or negative control plasmid. Cells were cultured in six-well plate to 50–60% confluence overnight, after which 2 tubes containing 125 μL of opti-MEM medium, and one was added with Lipo-3000 (7.5 μl, Lipofectamine^®^ 3,000 reagent, Invitrogen, United States) and mixed slowly, another was adden with 10 μl of P3000 and 5 μg of plasmid and mixed slowly. The two plasmids were added and mixed in the tube. The contents of the second tube with the P3000 and plasmids were transferred into the first tube and mixed slowly. The mixture was incubated for 5 min at room temperature. Finally, the mixture was slowly added to the six-well plate and incubated for 48 h. All of the plasmids were purchased from Genechem (Shanghai, China). Transfection efficiency was detected with western blot.

### Western Blotting

Total protein of cells and heart tissues were extracted and quantified with BCA Protein Assay kit (Beyotime, Shanghai, China), separated by SDS-PAGE, and then transferred to a PVDF membrane. Each membrane was blocked for 1.5 h at room temperature in 5% non-fat milk, followed by incubation overnight at 4°C with the primary antibodies against NLRP3 (1:1000, abcam, ab263899, United Kingdom), apoptosis associated speck like protein containing a CARD (ASC) (1:500, abcam, ab180799, United Kingdom), Caspase-1 (1:1000, Immunoway,YT5743, United States), cleaved-Caspase-1 (1:1000, abcam, ab179515 United States), YAP1 (1:2000, proteintech, 66900-1-Ig, China), phosphor-YAP1 (ser127) (1:1000, cell signaling technology, 13008s, United States), YAP1 (1:2000, proteintech, 66900-1-Ig, China), phosphor-YAP1 (ser127), LATS (1:1000, proteintech, 17049-1-AP, China), phosphor-LATS1 (Thr1079/1041) (1:1000, immunoway, YP1222, United States), and α-tubulin (1:2000, abcam, ab176560, United Kingdom). The membrence then incubated with the horseradish peroxidase (HRP)-labeled goat anti-rabbit and goat anti-mouse secondary antibodies for 1 h at room temperature. And the expression of protiens was visualized using enhanced chemilumi-nescence reagents (biosharp, BL520A, China).

### ELISA

The levels of folic acid, IL-1β and IL-18 in cell culture supernatants were detected using specific ELISA kits (Elabscience Biotechnology, Wuhan, China) according to the manufacturer’s instructions.

### Animals

Male C57BL/6 mice were fed with high fat diet (HFD; fat 60, protein 20, carbohydrate 20%, Greisway Biotechnology Co., Ltd. Suzhou, China) combined with 50 mg/kg streptozotocin (sigma, 18883664, Germany) intraperitoneally to establish a type 2 diabetes model, and normal group fed with ordinary diet. The successfully modeled mice were divided into two groups according to random number table: HFD + STZ group (n = 6) and folic acid treated group (n = 6, 8 mg/kg folic acid was given by gavage every 2 days), continue to feed HFD. The corresponding dose of PBS was given in HFD + STZ group. After 8 weeks of administration, the body weight, blood glucose were detected, and the cardiac tissues were harvest for protein detection. All experiments were performed according to the Guidelines of Animal Experiments from the Committee of Medical Ethics at the National Health Department of China and were approved by the Ethics Committee of Suzhou Science and Technology town Hospital.

### Statistical Analysis

The moderate t-test was used to identify DEGs. The data were obtained from three independent experments for *in vitro* studies and six mice in every experimental group for *in vivo* studies. Data are presented as the mean ± SD. The GraphPad Prism seven statistical software (San Diego, CA, United States) was used to conduct statistical analyses. One-way ANOVA was used to compare the differences among three groups, followed by Tukey’s post-hoc test to determine the differences between groups. Two-way ANOVA has been used to analyze the difference for the data of blood glucose in *in vivo* experiments. *p* < 0.05 was considered to indicate a statistically significant difference.

## Results

### Overall Differential Gene Expression Profiles

A total of 35,028 cadiac mRNA expression profiles were downloaded from the GEO database, and 427 DEGs were identified in the T2DM group relative to the control, of which 195 and 232 were respectively upregulated and downregulated (absolute fold change≥2.0, *p* < 0.05). The top 20 most significant DEGs are shown in the heatmap in Supplementary Figure S1A. The volcano plot of the DEGs is shown in Supplementary Figure S1B.

### Drug-Gene Interaction

Drug-gene interaction analysis of the top 20 most significant DEG revealed two potential drugs targeting two genes (Interaction score>20). As shown in Table 1, TOLRESTAT targeted AKR1B10, whereas Folr2 was the target of folic acid.

**TABLE 1:** Drug-gene interaction analysis of the top 20 most significant DEGs (Interaction score>20).

Gene	Durg	Interaction score
AKR1B10	TOLRESTAT	23.92
FOLR2	FOLIC ACID	63.79

### Folic Acid Alleviates HGF-Induced Pyroptosis in H9C2 Cells

**FIGURE 1:**
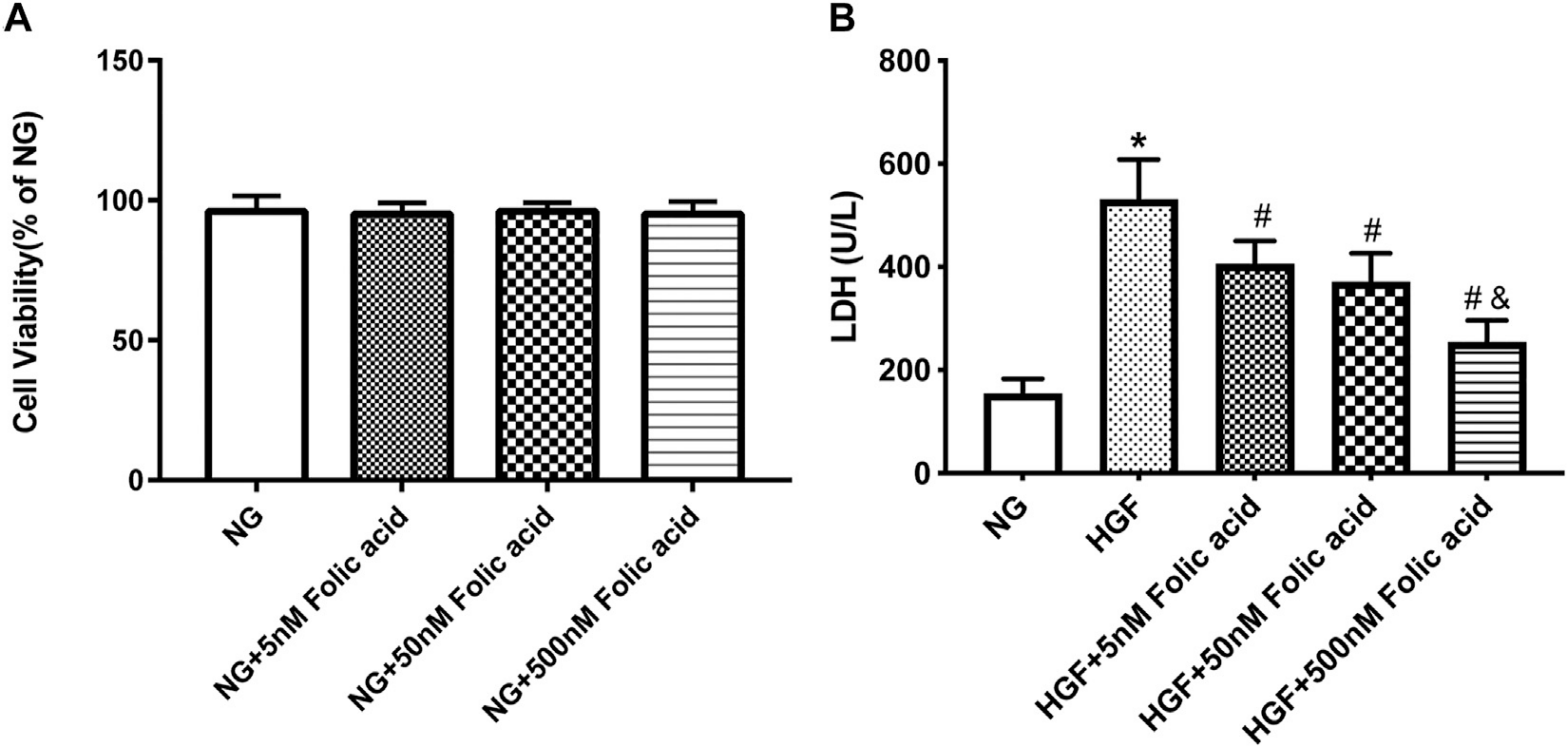
Effect of folic acid on cell viability in H9C2 cells. **(A)** Cell viabilities of folic acid on H9C2 cells. **(B)** The protective effects of 0–500 nM folic acid on the viability of H9C2 cells treated with HGF. *p < 0.05, compared with NG; ^#^ *p* < 0.05, compared with HGF; ^&^ *p* < 0.05, compared with HGF+50 nM folic acid.

**FIGURE 2:**
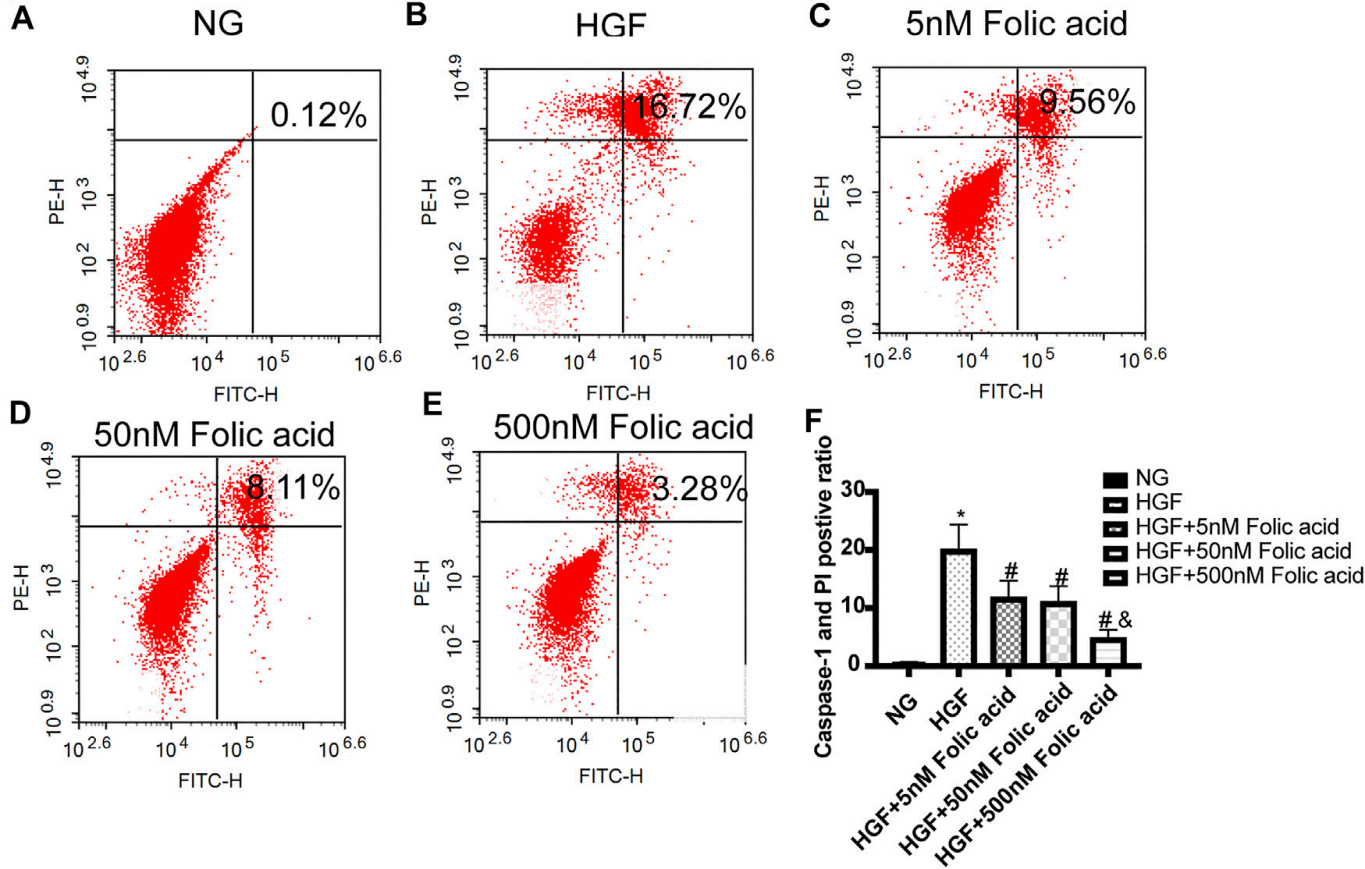
Effect of folic acid on the ratio of pyroptotic H9C2 cells. **(A–E)** Representive flow cytometry plots showing percentage of pyroptotic H9C2 cells treated with NG, HGF, 5–500 nM folic acid and HGF **(F)** Percentage of Annexin V and 7-AAD positive cells. ^*^ *p* < 0.05, compared with NG; ^#^ *p* < 0.05, compared with HGF; ^&^ *p* < 0.05, compared with HGF+50 nM folic acid.

**FIGURE 3:**
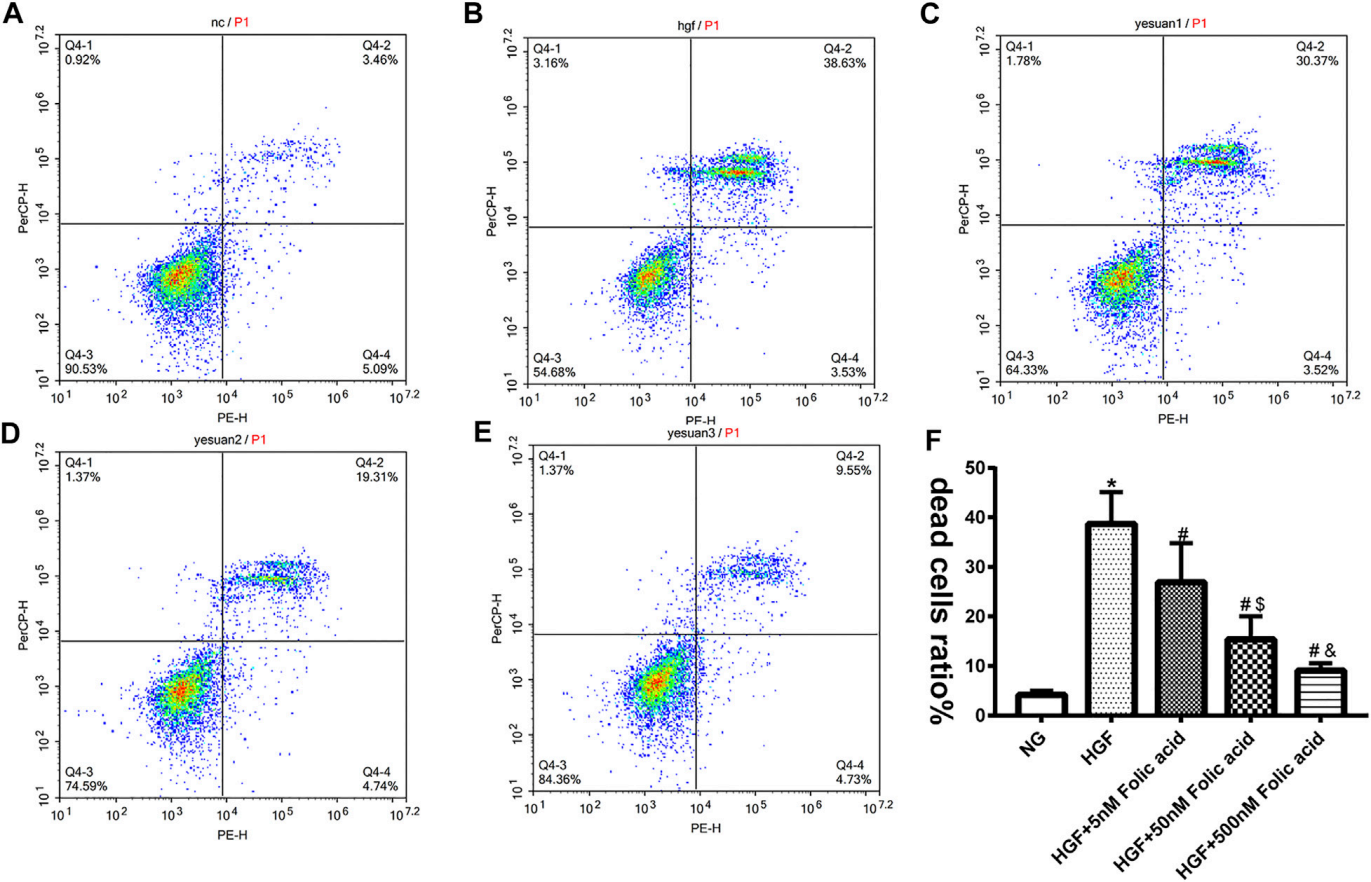
Effect of folic acid on the ratio of dead cells. **(A–E)** Representive flow cytometry plots showing percentage of dead cells treated with NG, HGF, 5–500 nM folic acid and HGF **(F)** Percentage of Annexin V and 7-AAD positive cells. ^*^ *p* < 0.05, compared with NG; ^#^ *p* < 0.05, compared with HGF; ^&^ *p* < 0.05, compared with HGF+50 nM folic acid.

**FIGURE 4:**
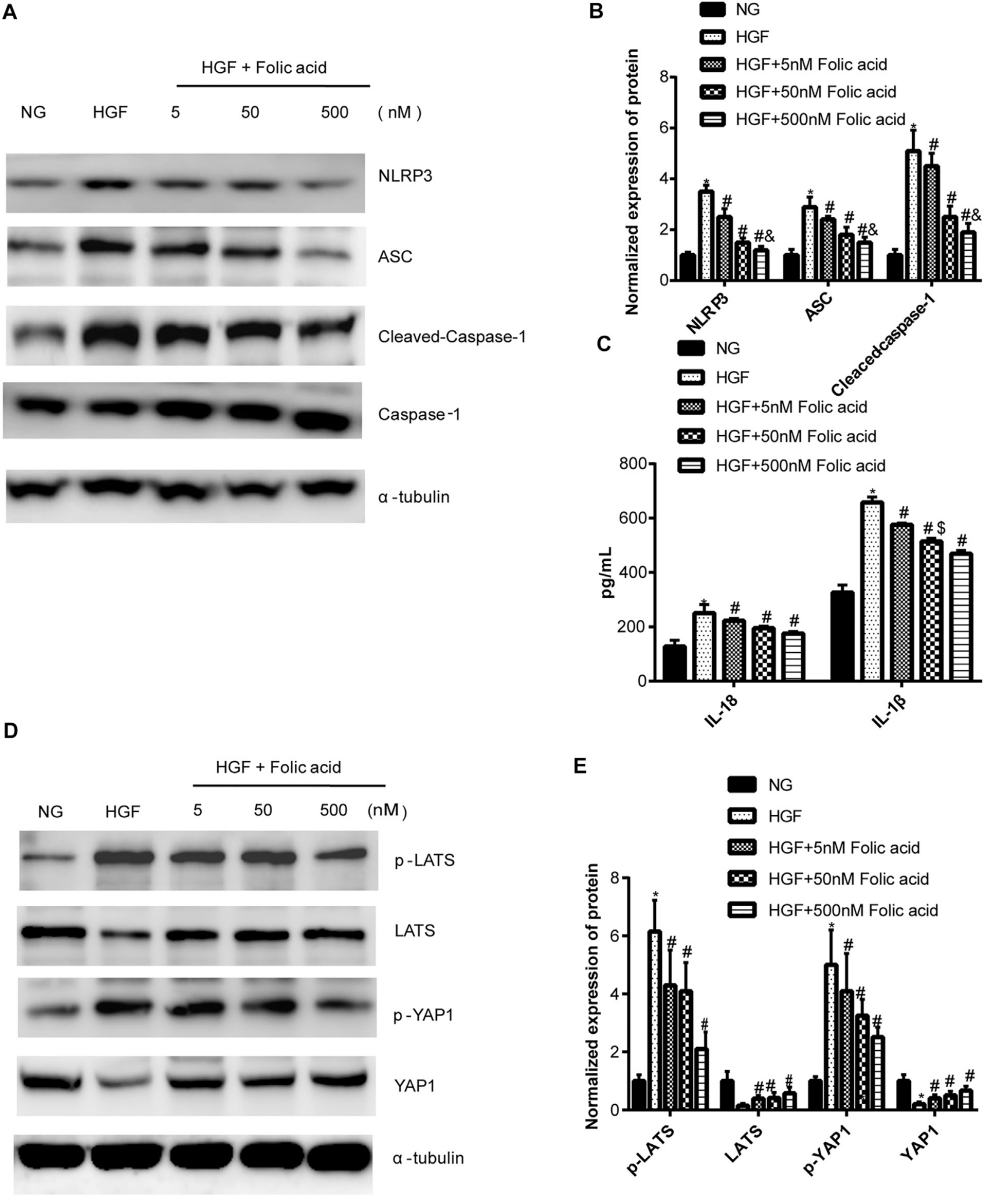
Effect of folic acid on the pyroptosis and Hippo signaling pathway. **(A)** Representative immunoblot showing expression levels of NLRP3, ACS, Cleaved-Caspase-1 and Caspase-1. **(B)** Quantification of NLRP3, ACS, Cleaved-Caspase-1 and Caspase-1 with α-tubulin as the loading control. **(C)** IL-1β and IL-18 levels in the cell supernatant. **(D)** Representative immunoblot showing expression levels of YAP1 and p-YAP1. **(E)** Quantification of YAP1 and p-YAP1 with α-tubulin as the loading control. ^*^ *p* < 0.05, compared with NG; ^#^ *p* < 0.05, compared with HGF; ^&^ *p* < 0.05, compared with HGF+50 nM folic acid.

As shown in Figure 1A, folic acid did not have any toxic effects on the H9C2 cells within the concentration range of 0–500 nM, while it showed protective effect on the H9C2 cells treated with HGF (Figure 1B) at 5, 50 and 500 nM. Furthermore, HGF treatment significantly increased the proportion of pyroptotic cells to15.8 ± 6.1% (Figures 2A,B, *p < 0.05, HGF vs NG), which was decreased to 11.71 ± 4.5, 10.1 ± 4.1 and 6.1 ± 2.9% by 5, 50 and 500 μM folic acid respectively (Figures 2C–E, ^#^ *p* < 0.05, compared to HGF; ^$^ *p* < 0.05, compared to HGF+5 nM folic acid; ^&^ *p* < 0.05, compared to HGF+50 nM folic acid). The results of Annexin V and 7-AAD staining also showed a protective effect, HGF treatment significantly increased the proportion of dead cells to 38.7 ± 6.4% (Figures 3A,B, ^*^ *p* < 0.05, HGF vs NG), which was decreased to 26.9 ± 7.9, 15.4 ± 4.6 and 9.1 ± 1.1% by 5, 50 and 500 μM folic acid respectively (Figures 3C–E, ^#^ *p* < 0.05, compared to HGF; ^$^ *p* < 0.05, compared to HGF+5 nM folic acid; ^&^ *p* < 0.05, compared to HGF+50 nM folic acid). Consistent with above results, HGF conditions also increased the expression of the NLRP3 inflammasome components ASC, Cleaved-caspase-1 and caspase-1, which comprise the main pathway of pyroptosis and inflammation. The increased expression of NLRP3 is an indication of pyroptosis priming. Folic acid supplementation markedly decreased the levels of the pyroptotic factors (Figures 4A,B, *p < 0.05, HGF vs NG; #*p* < 0.05, compared to HGF; &*p* < 0.05, compared to HGF+50 nM folic acid). Finally, folic acid significantly decreased the secreted levels of IL-1β and IL-18 under HGF conditions (Figure 4C, ^*^ *p* < 0.05, HGF vs NG; ^#^ *p* < 0.05, compared to HGF; ^&^ *p* < 0.05, compared to HGF+50 nM folic acid).

### Folic Acid Alleviates HGF-Induced Cardiomyocyte Injury Through the Hippo Pathway

**FIGURE 5:**
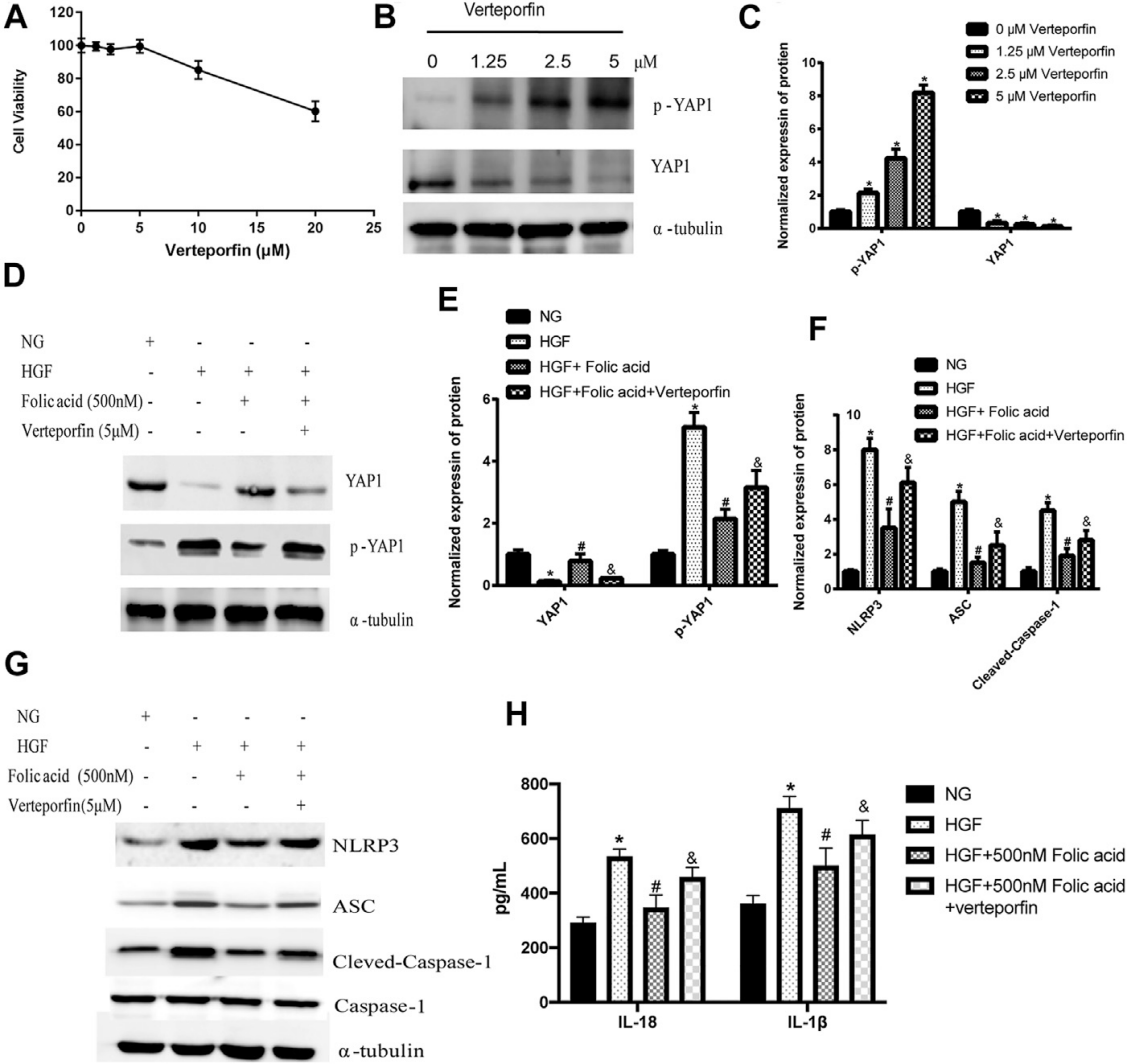
Verteporfin decreased the protective effect of folic acid on H9C2 cells by inhibiting YAP expression. **(A)** The effect of verteporfin on the percentage of viable H9C2 cells. **(B)** Representative immunoblot showing expression levels of YAP and p-YAP in H9C2 cells treated with verteporfin. **(D, G)** Representative immunoblot showing levels of NLRP3, ACS, Cleaved-Caspase-1, Caspase-1, YAP and p-YAP. **(H)** IL-1β and IL-18 levels in the cell supernatant. **(C, E, F)** Quantification of YAP1, p-YAP1, NLRP3, ACS, with α-tubulin as the loading control, for Cleaved-caspase-1, Caspase-1 as the loading control. ^*^ *p* < 0.05, compared with NG; ^#^ *p* < 0.05, compared with HGF, ^&^ *p* < 0.05, compared with HGF + Folic acid.

As shown in Figure 4D, HGF induced phosphorylation YAP and LATS1, and degradation of YAP and LATS1, which was abrogated by folic acid (Figure 4G, ^*^ *p* < 0.05, HGF vs NG; ^#^ *p* < 0.05, compared to HGF; ^&^ *p* < 0.05, compared to HGF+50 nM folic acid). Furthermore, the YAP inhibitor verteporfin downregulated YAP expression (Figures 5A,B) and neutralized the protective effect of folic acid (500 nM) on cardiomyocytes under HGF condition by upregulating NLRP3, ASC and Cleaved-caspase-1. In addition, IL-1β and IL-18 secretion was also significantly enhanced by verteporfin (Figures 5D,G,H). H9C2 cells were treated with 5 μM verteporfin, and the result showed that 5 μM verteporfin did not inhibited the expression of NLRP3 (Supplementary Figure S2).

**FIGURE 6:**
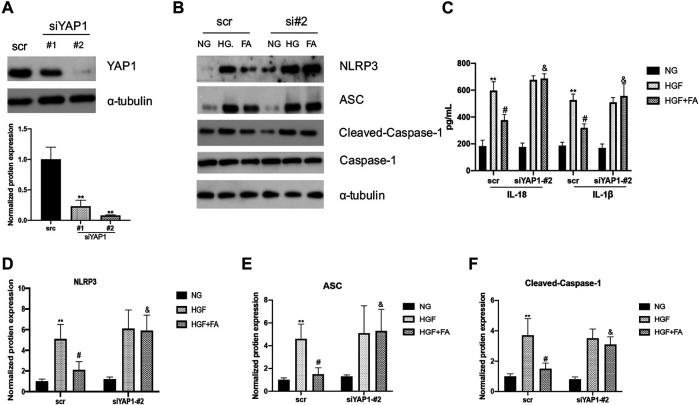
The anti-pyroptosis activity of folic acid was depended on YAP1. **(A)** siRNA significantly inhibits the expression of YAP1. **(B)** Representative immunoblot levels of NLRP3, ACS, Cleaved-Caspase-1, Caspase-1 in scr and siYAP1 H9C2 cells treated with different medium. **(C)** IL-1β and IL-18 levels in the cell supernatant. **(D–F)** Quantification of NLRP3, ACS, with α-tubulin as the loading control; for Cleaved-caspase-1, Caspase-1 as the loading control. **p < 0.01, compared with NG treated scr cells; #*p* < 0.01, compared with HGF treated scr cells, &*p* < 0.05, compared with HGF+ 500 nM folic acid treated siYAP1 cells.

SiRNA was used to inhibit the expression of YAP1 in H9C2 cells and assess the potential role of YAP1 in folic acid-mediated anti-pyroptosis. siYAP1 plasmid successfully inhibited the expression of YAP1 (Figure 6A, ^**^ *p* < 0.01, compared to scr group). Folic acid significantly inhibited the expression of NLRP3, ASC, Cleaved Caspase-1, IL-18 and IL-1β in HGF-treated negative control (scr) cells, but did not affect the expression of these genes in the group of siYAP1 cells (Figures 6B–F, ^**^ *p* < 0.01, compared with NG treated scr cells; #*p* < 0.01, compared with HGF treated scr cells, ^&^ *p* < 0.05, compared with HGF+ 500 nM folic acid treated siYAP1 cells).

### Folic Acid Alleviates HFD + STZ -Induced Cardiomyocyte Injury *in vivo*

**FIGURE 7:**
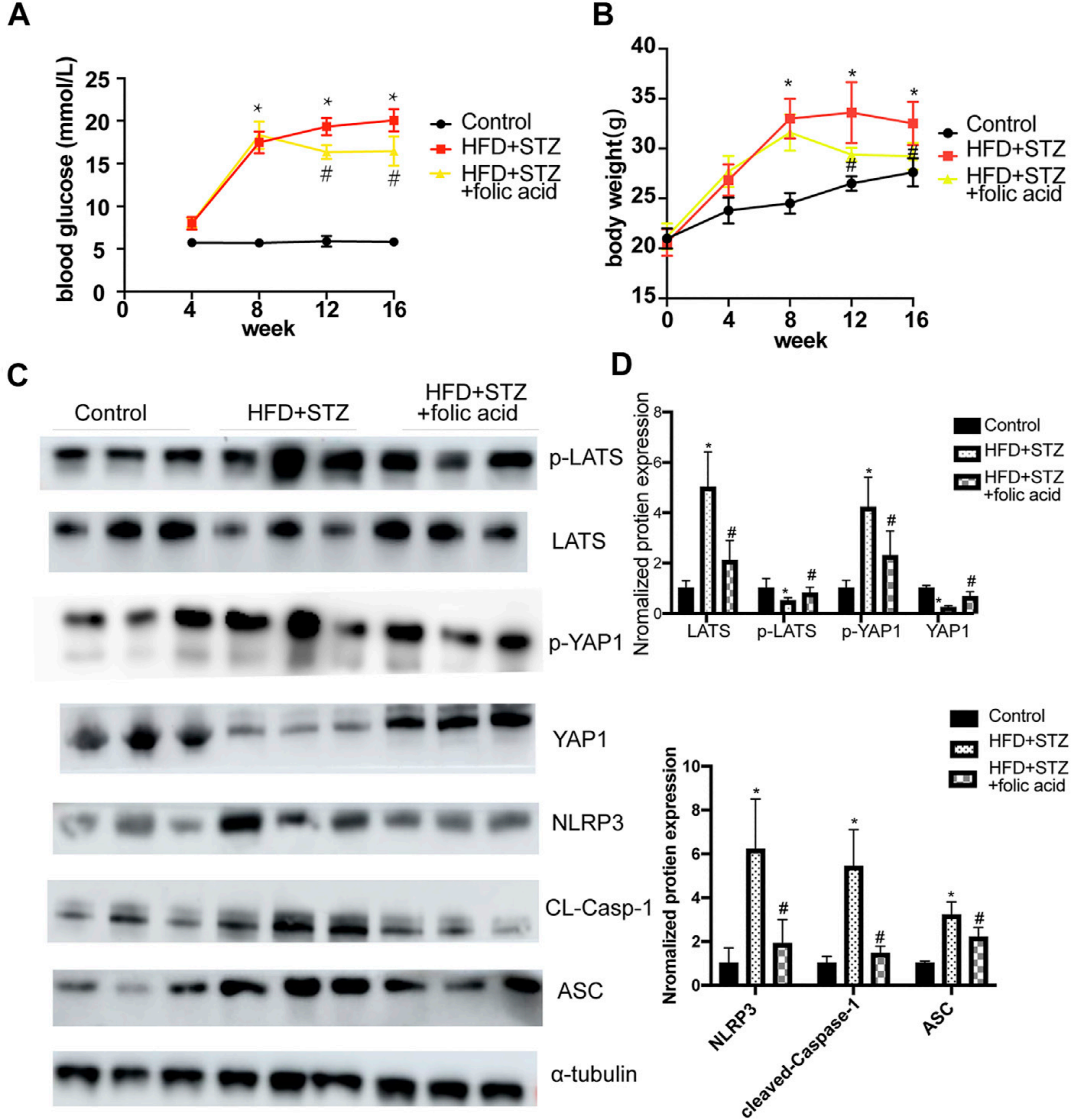
Folic acid reduces myocardial pyroptosis by inhibiting activation of Hippo signaling pathway in HFD + STZ mice. **(A)** Blood glucose curve; **(B)** body weight curve; **(C)** Representative immunoblot showing levels of NLRP3, ACS, Cleaved-Caspase-1, LATS, p-LATS, YAP and p-YAP; **(D)** Quantification of NLRP3, ACS, Cleaved-caspase-1, LATS, p-LATS, YAP1, p-YAP1, α-tubulin was used as the loading control. ^*^ *p* < 0.05, compared with control group; ^#^ *p* < 0.05, compared with HFD + STZ group. CON: normal diet mice; HFD + STZ: HFD + STZ mice treated with PBS; HFD + STZ + folic acid: HFD + STZ mice treated with folic acid.

A T2DM mouse model was established through a HGF diet combined with STZ intraperitoneal injection, and used folic acid (8 mg/kg) or corresponding solvents for 8 weeks. Folic acid could partially inhibit the body weight and blood glucose level of HFD + STZ mice (Figures 7A,B). Subsequently, the expression of pyroptosis-related proteins was detected by western blot, and the results showed that 8 mg/kg folic acid treatment could significantly inhibit the expression of pyroptosis-related proteins NLRP3, cleaved-caspase-1 and ASC. In addition, Hippo signaling pathway related protiens were also detected by western blot (Figure 7C), consistent with the results in *in vitro* experiments, Hippo signaling pathway was actived in HFD + STZ mice heart, and inhibited by folic acid treatment.

## Discussion

In this study, we have identified folic acid might be a putative drug for treating DCM through bioinformatics analysis, and experimentally proved that folic acid alleviated T2DM induced pyroptosis of cardiomyocytes by reducing NLRP3 inflammasome via the Hippo signaling pathway.

To verify the key the potential drugs, we have clarified 427 aberrantly expressed mRNAs in DCM rat through the Drug-Gene Interaction database. Drug-gene interaction indicated folic acid may be a potential drug targeted to Folr2. By binding to folate receptor (Folr), folic acid promotes proliferation of porcine pancreatic stem cells into insulin-secreting cells (Yang et al., 2021).

The complications of T2DM significantly affect the patient survival and quality of life. The cardiovascular complications are the leading causes of death among T2DM patients (Jain et al., 2020). The early subclinical stage of DCM is insidious and characterized by left ventricular hypertrophy, fibrosis and abnormal cell signaling (Wu et al., 2019), which progress in the later stages to extensive fibrosis, reduced myocardial compliance and diastolic dysfunction, eventually leading to heart failure, cardiogenic shock or arrhythmia (Xia et al., 2020). We modeled DCM *in vitro* by culturing the H9C2 cardiomyoblasts under HGF conditions, which significantly increased LDH release and pyroptosis that are indicative of cellular damage.

Although the protective effects of folic acid in diabetic complications have been reported previously (Ebaid et al., 2020), its potential cardioprotective role in T2DM is largely unknown. Folic acid improved cardiac function in sucrose-fed insulin resistant mice by ablating CaMKII phosphorylation (Roe et al., 2013), and rescued the vascular inflammatory response to high homocysteine levels in T2DM mice (Malaguarnera, et al., 2015). After 4 weeks of folic acid supplementation, the activity of antioxidant enzymes and malate dehydrogenase (MDH) in the heart tissues, as well as blood glucose levels, decreased in diabetic mice (Fang et al., 2019). NLRP3 inflammasome activation is a key step in pyroptosis and indication of pyroptosis priming (Yu et al., 2020). In our study as well, folic acid treatment significantly inhibited the expression of pyroptosis-related proteins NLRP3, cleaved-caspase-1 and ASC. It demonstrated that folic acid rescued cardiomyocytes from HGF-induced damage in a dose-dependent manner and decreased pyroptosis, the *in vivo* study also showed an decreased blood glucose levels, body weight and pyroptosis after folic acid treatment, indicating that folic acid supplementation is potentially beneficial for diabetes patients with cardiomyopathies.

The Hippo signaling pathway plays an important role in T2DM-related cardiac disease, and its core components MST and YAP are involved in diabetic progression (Liu, et al., 2020; Hu, et al., 2018). A studiy showed that the cardioprotective effect of folic acid was likely mediated via the Hippo signaling pathway (Wang, et al., 2018). The endothelial-specific Mst1 transgenic mice exhibited worse cardiac function and increased insulin resistance compared to the non-transgenic diabetic controls (Hu, et al., 2018). The cardiomyocytes ingested the exosomal MST1 secreted by endothelial cells under high glucose condition, which enhanced their apoptosis rates (J Hu et al., 2018). YAP1 is also associated with T2DM-induced myocardial fibrosis (Liu, et al., 2020) and renal interstitial fibrogenesis (J Chen J. et al., 2020). MST1/2 and LATS1/2 were down-regulated and showed increased phosphorylation levels during cardiomyocyte injury, which inhibited the nuclear translocation of YAP (Liu et al., 2019; Takaguri et al., 2020). Consistent with the above, we found that folic acid inhibited phosphorylation of YAP and LATS during the pyroptosis of H9C2 cells and T2DM mice, underscoring the involvement of the Hippo signaling pathway in T2DM-induced myocardial injury. The YAP inhibitor verteporfin (Futakuchi et al., 2018) abrogated the protective effects of folic acid on the cardiomyocytes, indicating that the Hippo signaling pathway is at least partially involved alleviating myocardial injury. Furthermore, knockdown of YAP1 did not completely improve the folic acid-induced alleviation of pyroptosis and death in HGF treated cells. These findings imply that folic acid may not only exert its protective effect on diabetic cardiomyopathy through YAP1 induced pyroptosis, but also regulate other manners of cell death.

In summary, folic acid treatment protected cardiomyocytes against HGF-induced pyroptosis by down-regulating the NLRP3 inflammasome and Hippo signaling pathway activation. Thus, folic acid can improve the cardiovascular complications of T2DM. However, our *in vitro and anmimal study* findings will have to be validated in clinical studies. In addition, the possible involvement of other signaling pathways in the protective effects of folic acid also need to be investigated.

## Data Availability

The datasets presented in this study can be found in online repositories. The names of the repository/repositories and accession number(s) can be found in the article/Supplementary Material.
