# Supplementary figures and images for: Folic Acid Alleviates High Glucose and Fat-Induced Pyroptosis via Inhibition of the Hippo Signal Pathway on H9C2 Cells
Source: Front Mol Biosci. 2021 Oct 7;8:698698. doi: 10.3389/fmolb.2021.698698 (PMC8529044; doi:10.3389/fmolb.2021.698698)

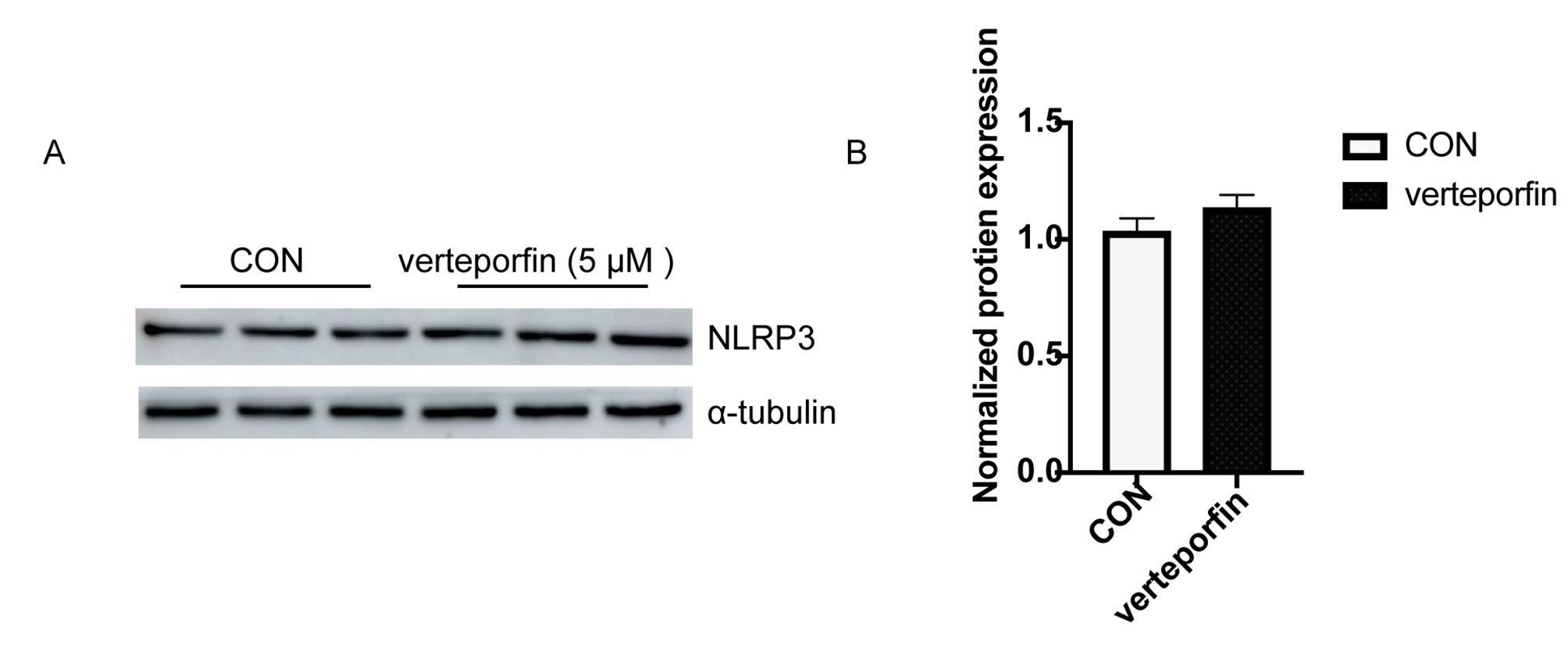

Supplement: Supplementary file 1 [file Image2.TIF]

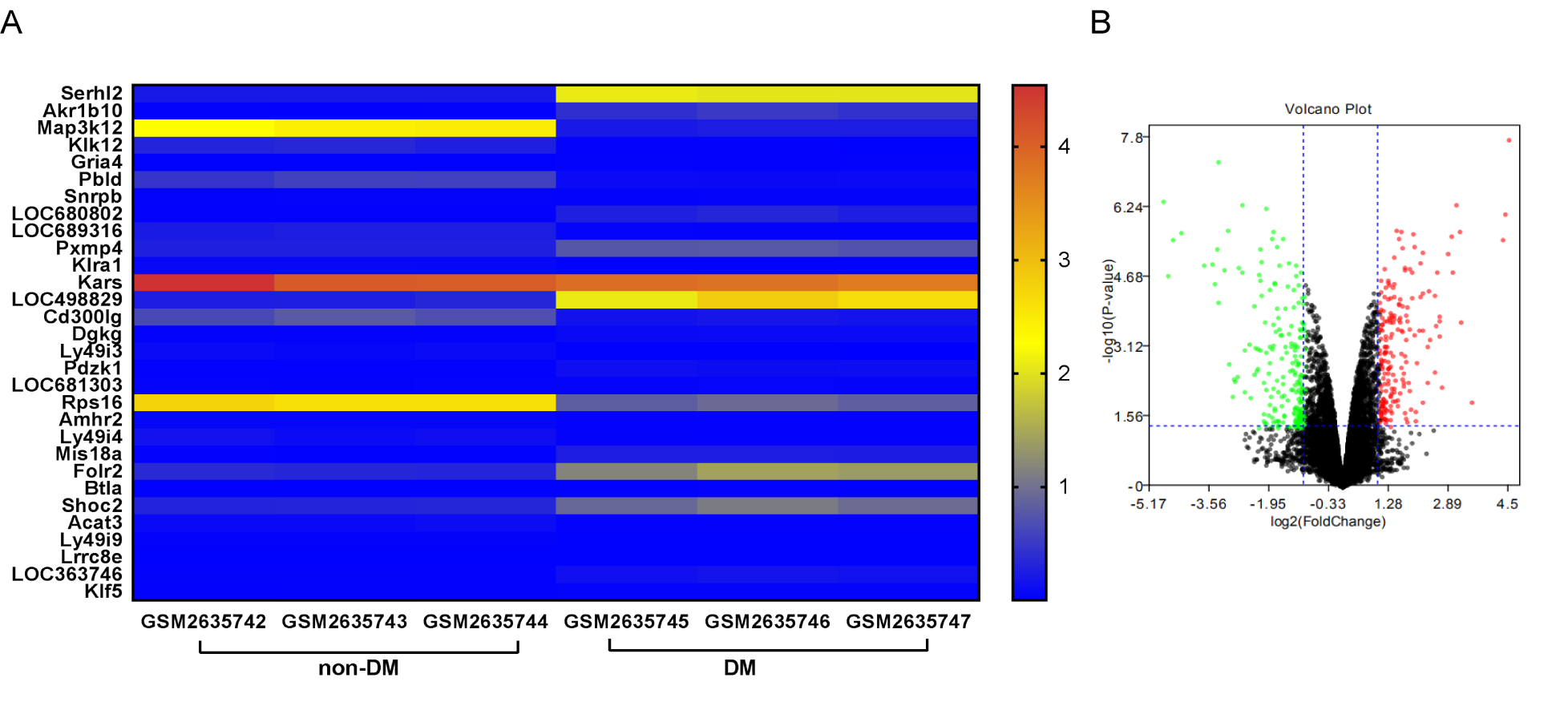

Supplement: Supplementary file 2 [file Image1.TIF]
